# The response of VEGF-stimulated endothelial cells to angiostatic molecules is substrate-dependent

**DOI:** 10.1186/1471-2121-6-38

**Published:** 2005-10-31

**Authors:** Christina L Addison, Jacques E Nör, Huijun Zhao, Stephanie A Linn, Peter J Polverini, Christie E Delaney

**Affiliations:** 1 Centre for Cancer Therapeutics, Ottawa Health Research Institute, 501 Smyth Rd., Ottawa Ontario, K1H 8L6, Canada; 2 Department of Cariology, Restorative Sciences and Endodontics, School of Dentistry, University of Michigan, 1011 North University Ave., Ann Arbor Michigan 48109-1078, USA; 3 Oral Medicine, Pathology and Oncology, School of Dentistry, University of Michigan, 1011 North University Ave., Ann Arbor Michigan 48109-1078, USA

## Abstract

**Background:**

The microenvironment surrounding cells can exert multiple effects on their biological responses. In particular the extracellular matrix surrounding cells can profoundly influence their behavior. It has been shown that the extracellular matrix composition in tumors is vastly different than that found in normal tissue with increased amounts of certain matrices such as collagen I. It has been previously demonstrated that VEGF stimulation of endothelial cells growing on type I collagen results in the induction of bcl-2 expression and enhanced endothelial cell survival. We sought to investigate whether this increased endothelial cell survival resulted in the failure of angiostatic molecules to inhibit angiogenesis.

**Results:**

We now demonstrate that VEGF-induced survival on collagen I impairs the ability of three known angiostatic molecules, TSP-1, IP-10 and endostatin to inhibit endothelial cell proliferation. Apoptosis of endothelial cells, growing on collagen I, induced by TSP-1 and IP-10 was also inhibited following VEGF stimulation. In contrast, endostatin induced apoptosis in these same cells. Further analysis determined that endostatin did not decrease the expression of bcl-2 nor did it increase activation of caspase-3 in the presence of VEGF. Alternatively, it appeared that in the presence of VEGF, endostatin induced the activation of caspase-8 in endothelial cells grown on collagen I. Furthermore, only endostatin had the ability to inhibit VEGF-induced sprout formation in collagen I gels.

**Conclusion:**

These data suggest that TSP-1, IP-10 and endostatin inhibit endothelial cells via different mechanisms and that only endostatin is effective in inhibiting angiogenic activities in the presence of collagen I. Our results suggest that the efficacy of angiostatic treatments may be impaired depending on the context of the extracellular matrix within the tumor environment and thus could impede the efficacy of angiostatic therapies.

## Background

The growth of tumors beyond 1 mm^3 ^is dependent on the induction of angiogenesis, defined as the growth of new blood vessels from preexisting vasculature [1]. The process of angiogenesis is regulated by a number of promoters (angiogenic) and inhibitors (angiostatic), and it is the balance of expression of these opposing molecules that ultimately dictates whether or not angiogenesis proceeds. There are a number of endogenous angiostatic inhibitors that have been actively investigated including thrombospondin, interferon-inducible protein 10 (IP-10) and endostatin. Thrombospondin (TSP) was initially identified as a human platelet derived protein [2] that played a key role in platelet aggregation [3,4]. It was subsequently shown to modulate the biological responses of endothelial cells [5,6], induce apoptosis of endothelial cells via a caspase-3 dependent mechanism [7-9] and inhibit pathological angiogenesis [10-13]. IP-10 is a CXC chemokine that is secreted by a variety of different cell types in response to interferon stimulation [14]. In addition to being a T-cell chemoattractant [15,16], IP-10 has been shown to be an inhibitor of angiogenesis [17-19] and tumor growth *in vivo *[19-21]. Endostatin is the 20 kDa C-terminal cleavage product of collagen XVIII that has been shown to have a number of anti-angiogenic properties including inhibition of endothelial cell proliferation [22] and migration [23,24], induction of endothelial cell apoptosis [25], and inhibition of tumor growth *in vivo *[22,26-28]. Although these angiostatic molecules have been shown to be efficacious in pre-clinical models, their success in clinical trials has been more limited. Part of this may be due to the fact that very little is understood about the mechanisms by which these molecules exert their biological effects and how their efficacy might be altered by different tumor microenvironments.

The extracellular matrix (ECM) composition in tumors is vastly different than that found in its normal tissue counterparts. Certain ECM proteins such as collagen I, fibronectin and tenascin C are increased, while the basement membrane proteins collagen IV and laminin are decreased in neoplastic as compared to normal breast tissue [29,30]. Previous data suggested that stimulation of human dermal microvascular endothelial cells (HDMEC) grown on collagen I with vascular endothelial growth factor (VEGF) resulted in the increased survival of these cells by a mechanism involving the upregulation of bcl-2 [31]. It has also been shown that increased endothelial cell survival following overexpression of bcl-2 was associated with enhanced tumorigenesis in a xenograft model of human tumorigenesis [32]. These observations suggested that increased endothelial cell survival might be modulated by extracellular matrix components such as collagen I, and may contribute to the progression of tumor growth and metastasis as a result of enhanced angiogenic potential within these tumors. Furthermore, this increased survival may render the endothelial cells more resistant to the inhibitory effects of certain angiostatic molecules, thus limiting their efficacy in certain tumor microenvironments. As collagen I is found to be overexpressed in tumor versus normal tissue of the breast [29,30], and is itself a substrate for attachment of integrins that can profoundly influence endothelial function [33-37], we therefore examined the ability of three known angiostatic molecules, TSP-1, IP-10 and endostatin to inhibit endothelial cell proliferation, induce endothelial cell apoptosis on both plastic and collagen I coated surfaces *in vitro *and to inhibit VEGF-induced endothelial cell tube formation in collagen I gels *in vitro*. We found that although TSP-1 and IP-10 could inhibit endothelial cell proliferation and induce apoptosis when HDMEC were cultured on plastic tissue culture dishes, their ability to inhibit these processes was impaired following culture of HDMEC on collagen I. We observed similar results when cells were exposed to endostatin in that it inhibited proliferation of HDMEC cultured on plastic, but failed to inhibit proliferation of HDMEC grown on collagen I. In contrast to the results observed with TSP-1 and IP-10, we found that endostatin retained the ability to induce apoptosis of HDMEC even in the presence of collagen I. Furthermore, only endostatin could inhibit the formation of VEGF-induced sprouts on collagen I gels *in vitro*, while TSP-1 and IP-10 remained ineffective in this model system. These data suggest that the context of the tumor matrix microenvironment may modulate the inhibitory activity of angiostatic molecules and this may have significant impact on the ability of these molecules to function as anti-angiogenic therapeutics clinically in certain tumor types.

## Results

### Growth on collagen I impairs the ability of TSP-1, IP-10 and endostatin to inhibit endothelial cell proliferation

**Figure 1:**
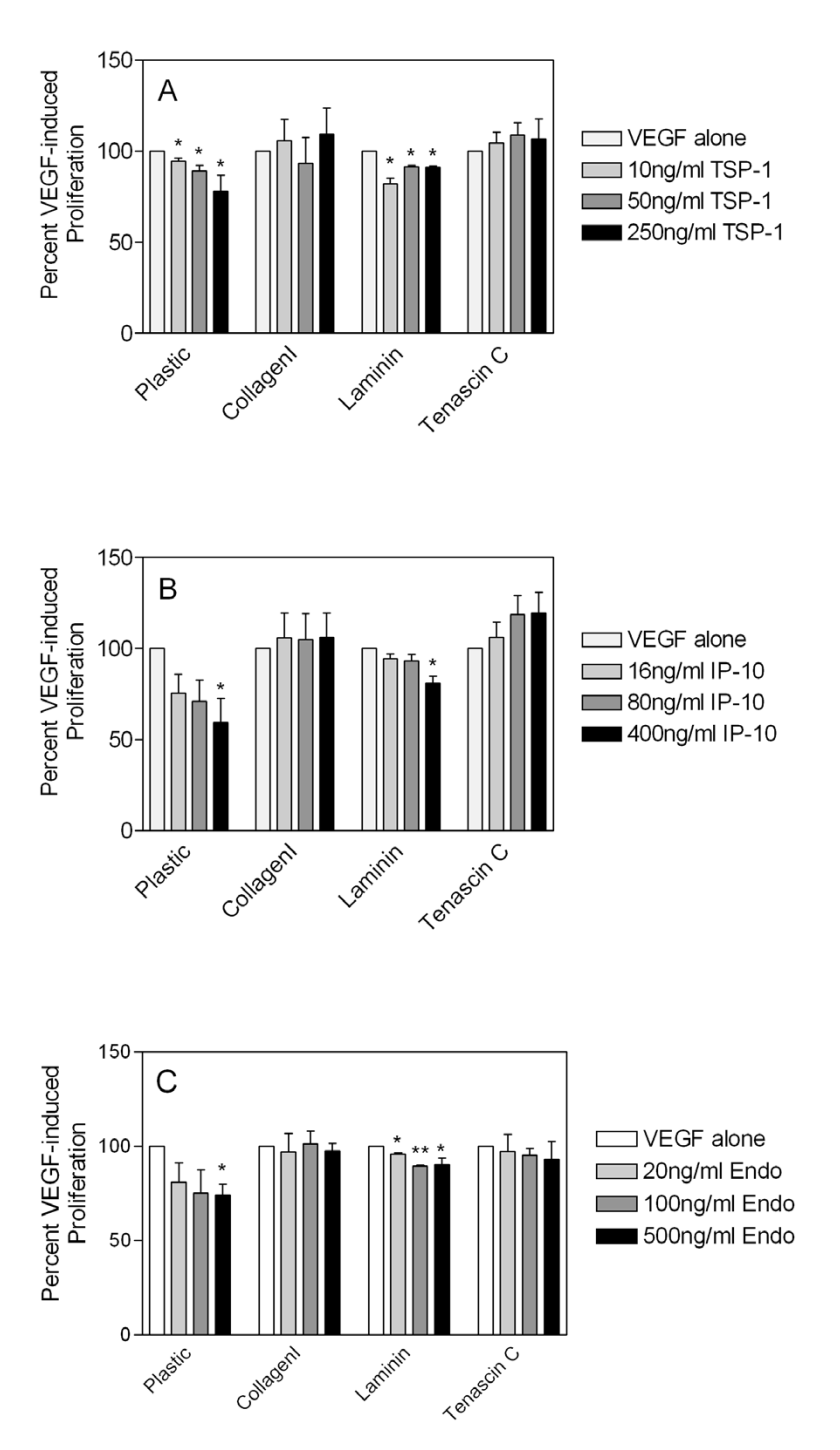
**Growth on tumor-associated matrices impairs the ability of TSP-1, IP-10, and endostatin to inhibit endothelial cell proliferation**. HDMEC were seeded onto plastic or dishes coated with either collagen I, laminin or tenascin C, starved and then stimulated with either 50 ng/ml VEGF alone or in combination with 10, 50 or 250 ng/ml TSP-1 (panel A), 16, 80 or 400 ng/ml IP-10 (panel B) or 20, 100 or 500 ng/ml recombinant endostatin (panel C) for 72 h. Adherent cells were harvested by trypsinization or collagenase treatment (in the case of collagen I coated dishes) and direct cell counts were determined by counting using a Coulter Counter. All bars represent the mean and standard error of triplicate dishes from three independent experiments (n = 9). Within each experiment all samples were normalized to VEGF-induced proliferation and statistical significance (* = p < 0.05, ** = p < 0.001) was determined using unpaired t-tests as compared to VEGF.

There is a significant body of literature demonstrating that the matrix microenvironment within tumors can be vastly different than that found in the normal tissue counterparts [29,30]. Furthermore, there is increasing evidence that "tumor-associated" ECM proteins may enhance the proliferation or survival of endothelial cells. Thus, tumor microenvironments may create a pro-angiogenic surrounding that impairs the ability of angiostatic molecules to inhibit endothelial cell growth and survival. We sought to determine if the angiostatic molecules TSP-1, IP-10 and endostatin could mediate the same inhibitory effects in endothelial cells when cells were cultured on plastic or on collagen I coated dishes. Collagen I was chosen as a "tumor-associated" ECM protein as its overexpression has been documented in a number of tumor types, and previous data suggested that growth of HDMEC on collagen I followed by stimulation with the angiogenic factor VEGF led to an increase in bcl-2 expression and enhanced survival of endothelial cells [31]. As these results suggested an increase in the survival potential of endothelial cells when cultured on collagen I, we initially investigated whether various angiostatic molecules could inhibit the proliferation of endothelial cells when cultured on tissue culture treated plastic dishes or collagen I coated dishes. Cells were starved overnight and then stimulated with 50 ng/ml VEGF (determined to be the optimal stimulatory dose of VEGF, data not shown) in combination with various doses of each inhibitor, and 72 h post-stimulation, cells were collected by trypsinization or collagenase treatment and the total number of cells per well was counted using a Coulter Counter. We observed a dose-dependent decrease in VEGF-induced HDMEC proliferation following addition of TSP-1 (Figure 1A), IP-10 (Figure 1B) and endostatin (Figure 1C) when cells were cultured on plastic. In contrast, all three angiostatic molecules failed to inhibit VEGF-induced proliferation when HDMEC were cultured on collagen I as a substrate (Figure 1). These results suggested that the ability of the various angiostatic molecules to inhibit VEGF-induced endothelial cell proliferation was impaired by growth of these cells on the matrix collagen I. As further confirmation, we examined the ability of the inhibitors to function following growth of HDMEC on the normal basement membrane ECM laminin, or on the tumor-associated ECM tenascin C. In these cases we saw statistically significant inhibition of cell proliferation by all three inhibitors when HDMEC were cultured on laminin while HDMEC were resistant to the inhibitory effects of the inhibitors following culture on tenascin C (Figure 1). These results suggest that ECM proteins associated with tumor growth, such as collagen I and tenascin C, enhance endothelial cell survival and impart a resistance to anti-angiostatic molecules.

**Figure 2:**
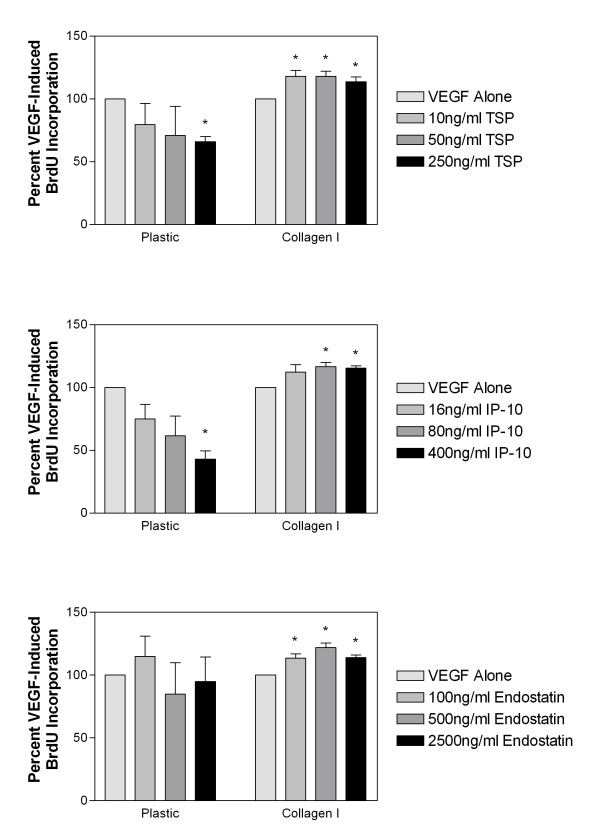
**Growth on collagen I impairs HDMEC proliferation as measured by BrdU incorporation**. HDMEC were seeded onto plastic or dishes coated with collagen I, starved and then stimulated with 50 ng/ml VEGF either alone or in combination with 10, 50 or 250 ng/ml TSP-1 (panel A), 16, 80 or 400 ng/ml IP-10 (panel B) or 100, 500 or 2500 ng/ml recombinant endostatin (panel C) for 48 h. BrdU labeling mixture was then added and the cells were incubated overnight at which time incorporated BrdU was quantitated using the BrdU Cell Proliferation ELISA (Roche). All bars represent the mean and standard error of triplicate samples. All samples were normalized to VEGF-induced BrdU incorporation and statistical significance (* = p < 0.05) was determined using unpaired t-tests as compared to VEGF.

We examined whether or not the apparent inhibition of proliferation as measured by direct cell counts was as a result of inhibition of cellular proliferation and replication or as a result of increased apoptosis of cells. To address this issue, we directly measured DNA replication by BrdU-incorporation assays. Cells were plated in 96-well microtiter plates, starved overnight in MCDB 131 supplemented with 1% fetal bovine serum (FBS), and then stimulated with VEGF either alone or in combination with various doses of TSP-1, IP-10 or endostatin. BrdU labeling mixture was then added 48 h post-stimulation and cells were incubated overnight. The amount of incorporated BrdU was then determined using the cell proliferation BrdU ELISA according to the manufacturer's directions. As was seen previously using direct cell counts, we saw statistically significant dose-dependent decreases in VEGF-induced endothelial cell proliferation as measured by BrdU incorporation following treatment with TSP-1 (Figure 2A) and IP-10 (Figure 2B). We also observed a trend in the reduction of BrdU incorporation following treatment with endostatin (Figure 2C), however this did not reach statistical significance. As seen previously, following growth of endothelial cells on collagen I, all three molecules were unable to inhibit VEGF-induced proliferation of HDMEC (Figure 2). These data suggest that one of the main mechanisms of inhibition of angiogenesis by TSP-1 and IP-10 is inhibition of endothelial cell proliferation, and that this mechanism is impaired by growth of cells on collagen I.

### Collagen I impairs endothelial cell apoptosis induced by TSP-1 and IP-10, but not by endostatin

**Figure 3:**
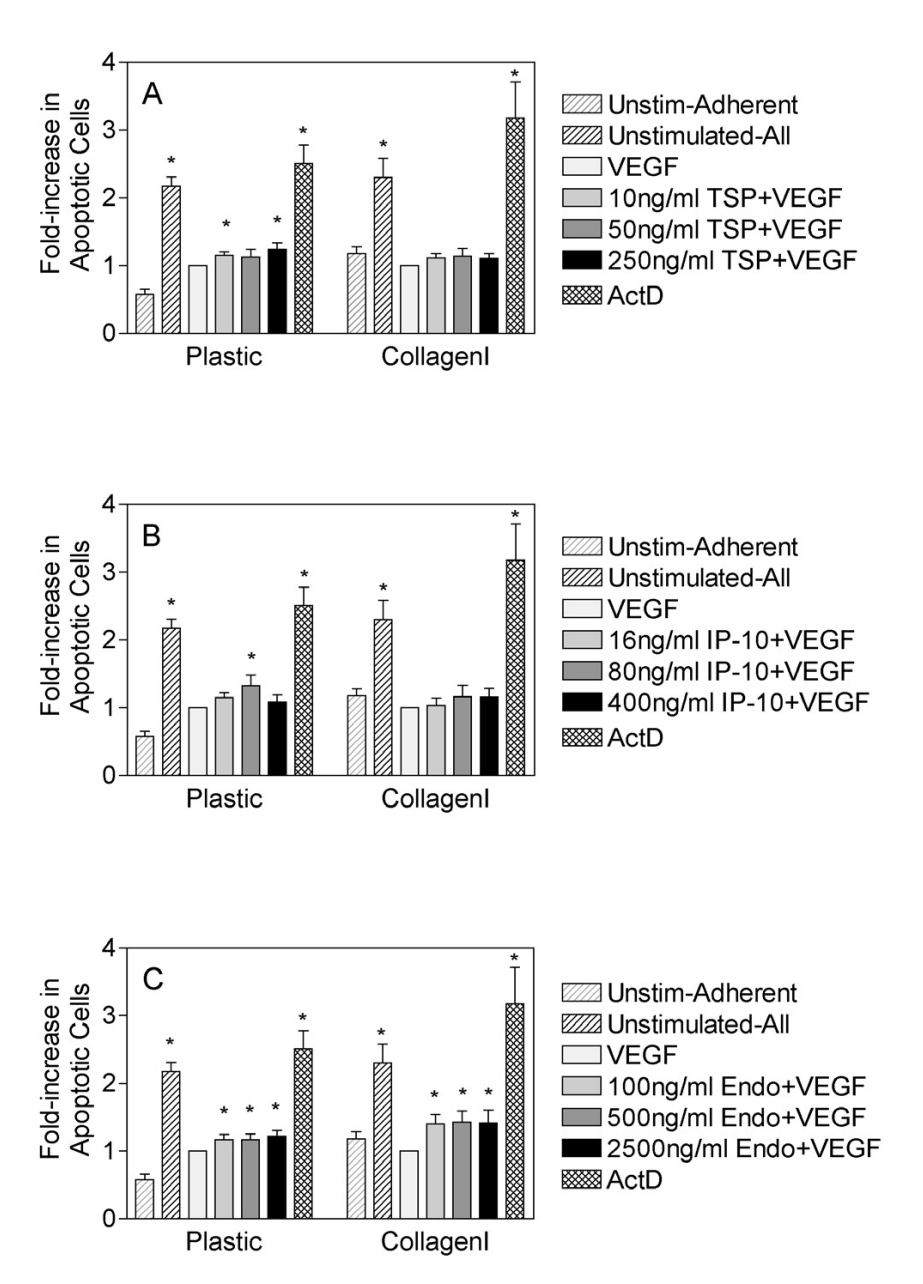
**Growth on collagen I impairs the ability of TSP-1 and IP-10, but not endostatin to induce apoptosis of HDMEC**. HDMEC were seeded onto plastic or collagen I coated tissue culture dishes, monolayers were washed to remove nonadherent cells, and subsequently adherent cells were stimulated with either 50 ng/ml VEGF alone or in combination with 10, 50 or 250 ng/ml TSP-1 (panel A), 16, 80 or 400 ng/ml IP-10 (panel B) or 100, 500 or 2500 ng/ml recombinant endostatin (panel C) for 60 h. Non-adherent and adherent cells were then collected, washed twice with PBS, and permeabilized in 70% ethanol for a minimum of 24 h prior to staining with propidium iodide. The percentage of apoptotic cells was determined following identification of the subG1 population of cells using FACS analysis. The adherent and nonadherent populations of unstimulated cells were included as a control for the basal level of apoptosis of HDMEC (unstimulated-all) and an adherent-only population of unstimulated cells was included to demonstrate the surviving fraction of untreated HDMEC (unstim-adherent). Cells treated with 50 nM of actinomycin D were included as a positive control for apoptosis. All samples were normalized to the levels of apoptosis observed following stimulation with 50 ng/ml VEGF alone. Bars are representative of the mean and standard error of duplicate dishes from three independent experiments (n = 6). Statistical significance was determined following analysis using unpaired t-tests as compared to VEGF (* = p < 0.05).

Many angiostatic molecules have been shown to induce apoptosis of endothelial cells and this has been proposed as one of the main mechanisms by which they inhibit angiogenesis [7-9]. Since we observed that growth of HDMEC on the tumor-associated matrices collagen I and tenascin C impaired the ability of the anti-angiogenic molecules to inhibit VEGF-induced proliferation, we next examined whether growth on collagen I influenced the ability of the angiostatic molecules to induce apoptosis of endothelial cells, by examining the proportion of cells in sub-G1 following staining with propidium iodide as a measure of apoptotic cells. For both HDMEC grown on plastic or collagen I, we observed a statistically significant reduction in the number of apoptotic cells following treatment with VEGF (Figure 3), thus demonstrating its ability to enhance survival of endothelial cells as has been previously documented. It should be noted that unstimulated HDMEC have a high level of apoptosis following 62 h in culture without media changes. This is a result of serum deprivation that is overcome by the addition of VEGF to the culture media in the same time frame. Similar to our results with HDMEC proliferation, we found that TSP-1 could partially overcome the protective effects of VEGF and induced a statistically significant induction of apoptosis when cells were cultured on plastic (Figure 3A). In contrast, when HDMEC were cultured on collagen I and treated with VEGF in combination with TSP-1, we observed only a small but statistically insignificant increase in apoptosis (Figure 3A) suggesting that TSP-1 was ineffective at inducing apoptosis of endothelial cells in the presence of collagen I. Similar results were observed following treatment of HDMEC with IP-10, where we observed statistically significant induction of apoptosis using 80 ng/ml of IP-10 when cells were cultured on plastic and stimulated with VEGF, and the failure of IP-10 to induce significant apoptosis when cells were cultured on collagen I in the presence of VEGF (Figure 3B). Surprisingly, we observed that endostatin significantly induced apoptosis of VEGF-stimulated HDMEC regardless of whether they were cultured on plastic or collagen I as a substrate (Figure 3C). These data suggest that even though all three of the inhibitors tested in these experiments have the capacity to induce endothelial cell apoptosis, they likely do so through different mechanisms that may or may not be affected by the substrate upon which the cells are cultured. Taken together with our data regarding BrdU incorporation, these data suggest that endostatin has greater effects on apoptosis of endothelial cells as opposed to their proliferation.

### The pro-apoptotic activity of endostatin is independent of bcl-2 expression

**Figure 4:**
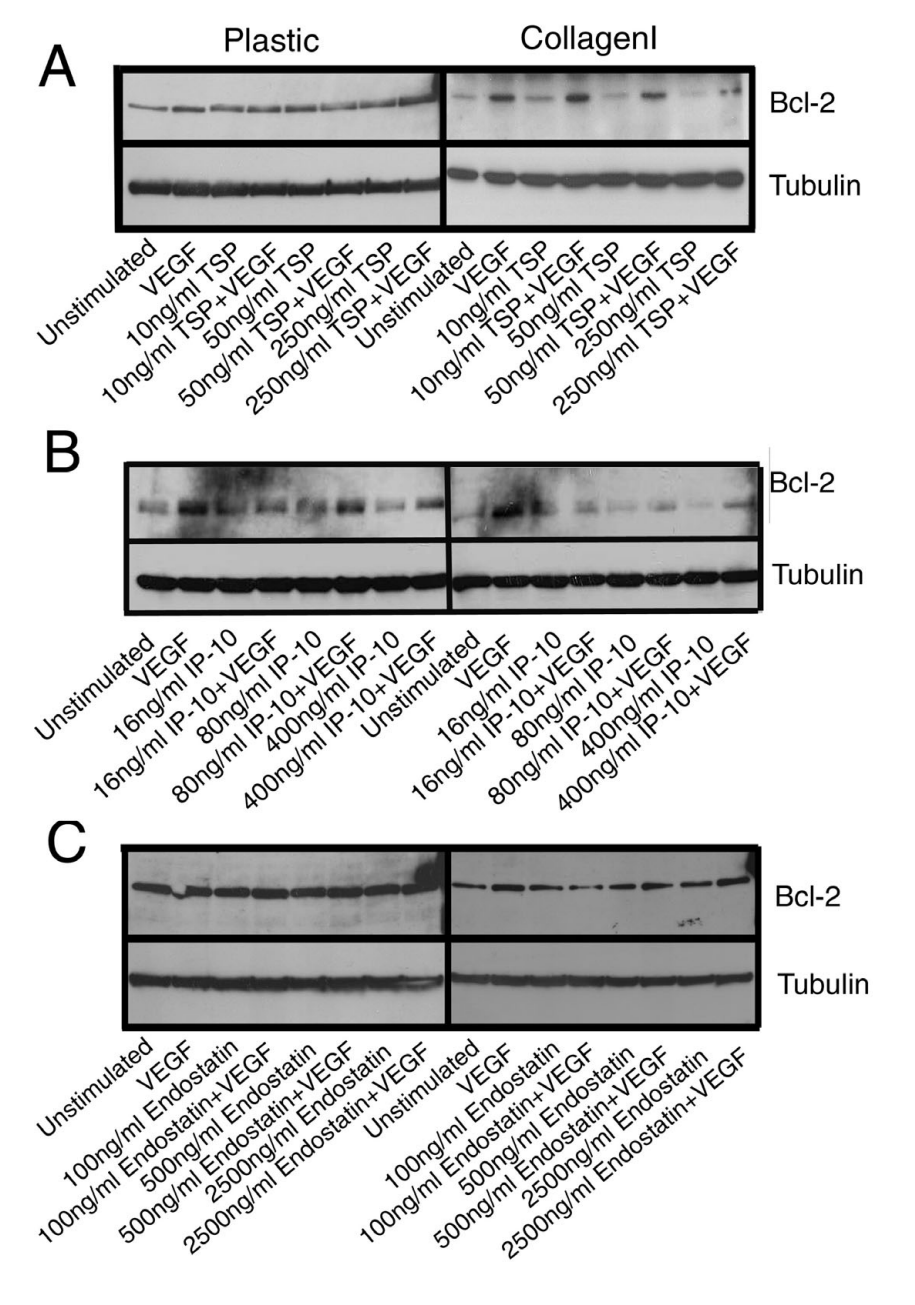
**HDMEC treated with TSP-1 and IP-10, but not endostatin show decreases in bcl-2 expression that is inhibited by concomitant VEGF stimulation**. HDMEC were seeded on either plastic or collagen I coated tissue culture dishes, and following removal of non-adherent cells by washing, were stimulated with either 50 ng/ml VEGF alone or in combination with 10, 50 or 250 ng/ml TSP-1 (panel A), 16, 80 or 400 ng/ml IP-10 (panel B), or 100, 500 or 2500 ng/ml recombinant endostatin (panel C) for 48 h. Cells were collected by trypsinization or collagenase treatment and total protein lysates were generated. Western blots for bcl-2 were performed as described in the methods section. Blots were stripped and reprobed for tubulin as a loading control.

To further elucidate the mechanism by which the angiostatic molecules induced apoptosis, and whether these mechanisms were affected by the presence of collagen I, we examined the ability of angiostatic molecules to modulate the levels of the anti-apoptotic protein bcl-2 which we had previously found to be upregulated in HDMEC on collagen I following VEGF treatment [31], and the activation of caspase-3 as one of the main mediators of apoptosis in endothelial cells. HDMEC cultured on either plastic or collagen I substrates were stimulated with increasing concentrations of TSP-1, IP-10 or endostatin, alone or in combination with VEGF and total protein lysates were generated. Initially a time course was performed and it was determined that changes in the expression of the proteins of interest were maximal at 48 h post-stimulation (data not shown). As seen previously, we observed that VEGF stimulation of HDMEC on collagen I induced an increase in the level of bcl-2 protein as compared to unstimulated controls (Figure 4, right hand panels, lanes 1 and 2). A similar increase in bcl-2 levels was not observed following VEGF-stimulation of HDMEC cultured on plastic dishes (Figure 4, left hand panels, lanes 1 and 2). In fact, when total protein loading is taken into consideration, it appeared that the levels of bcl-2 in HDMEC grown on plastic dishes did not change following treatment with any of the angiostatic molecules either alone or in combination with VEGF (Figure 4, left hand panels). In contrast to the results observed following culture on plastic, we observed a dose-dependent decrease in the expression of bcl-2 following treatment with TSP-1 alone (Figure 4A, right hand panel, lanes 3, 5 and 7 as compared to lane 1) or IP-10 alone (Figure 4B, right hand panel, lanes 3, 5 and 7 as compared to lane 1) as compared to unstimulated HDMEC grown on collagen I. This decrease in bcl-2 expression in HDMEC grown on collagen I was also apparent for TSP-1 (Figure 4A right hand panel, lanes 4, 6 and 8 as compared to lane 2) and IP-10 (Figure 4B right hand panel, lanes 4, 6 and 8 as compared to lane 2) treatments in combination with VEGF, but only when the highest doses of angiostatic molecules were used. The observed decrease in bcl-2 expression could account for the small increases in endothelial cell apoptosis noted in HDMEC grown on collagen I following stimulation with TSP-1 or IP-10 (Figure 3A,B). With respect to stimulation of HDMEC on collagen I with endostatin, there was a slight decrease in bcl-2 expression following treatment with 100 ng/ml endostatin in combination with VEGF (Figure 4C, right hand panel, lane 4 as compared to lane 2) when HDMEC were grown on collagen I, however, no dose-dependent decrease in bcl-2 expression was observed on either plastic (Figure 4C, left hand panel) or collagen I (Figure 4C, right hand panel) in response to endostatin treatment. Thus, it is unlikely that changes in bcl-2 expression are the cause of the increase in apoptosis of HDMEC observed in response to endostatin treatment on either of these substrates at the higher doses of inhibitor. We also probed for bax expression levels in these same samples and observed that there was no change in bax protein regardless of the treatment used or upon which matrix the HDMEC were cultured (data not shown). Thus it would appear that VEGF induces increased survival of HDMEC on collagen I in part as a result of its ability to upregulate bcl-2. Moreover, the slight decreases in bcl-2 expression observed following treatment with TSP-1 or IP-10 in combination with VEGF are not sufficient to overcome this increased survival and fail to induce significant endothelial cell apoptosis when HDMEC are cultured on collagen I. In contrast, the ability of endostatin to overcome the VEGF-induced HDMEC survival on collagen I appeared to be independent of the levels of bcl-2 expression.

### Endostatin-induced endothelial cell apoptosis is independent of caspase-3 activation

**Figure 5:**
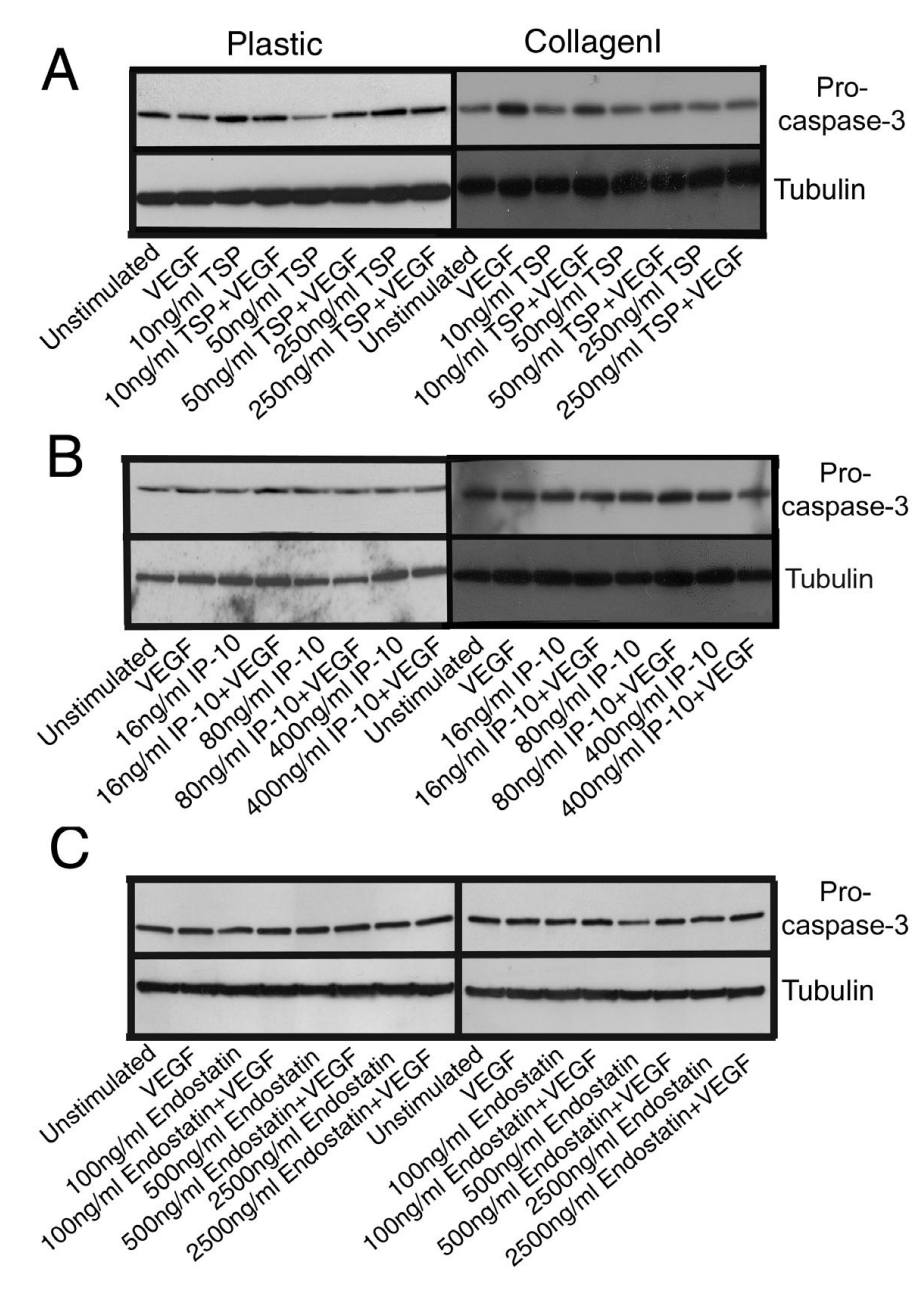
**HDMEC treated with TSP-1 and IP-10, but not endostatin show slight decreases in procaspase-3 expression**. HDMEC were seeded on either plastic or collagen I coated tissue culture dishes, and following removal of non-adherent cells by washing, were stimulated with either 50 ng/ml VEGF alone or in combination with 10, 50 or 250 ng/ml TSP-1 (panel A), 16, 80 or 400 ng/ml IP-10 (panel B), or 100, 500 or 2500 ng/ml recombinant endostatin (panel C) for 48 h. Cells were collected by trypsinization or collagenase treatment and total protein lysates were generated. Western blots for procaspase-3 were performed as described in materials and methods. Blots were stripped and reprobed for tubulin as a loading control.

The angiostatic molecules used in these studies demonstrated some capacity to induce apoptosis when HDMEC were cultured on plastic; however, only endostatin appeared able to induce apoptosis of HDMEC cultured on collagen I. To determine potential mechanisms of induction of apoptosis, we examined whether or not the angiostatic molecules in question led to the activation of caspase-3 when cells were cultured on either plastic or collagen I as a substrate. At high doses of TSP-1, we saw a decrease in procaspase-3, indicating cleavage of this protein into active caspase-3, following treatment of HDMEC cultured on plastic with 50 ng/ml TSP-1 as compared to unstimulated controls (Figure 5A, left hand panel, lane 5 as compared to lane 1). For all other treatment conditions of HDMEC cultured on plastic there did not appear to be cleavage or activation of procaspase-3, hence no reduction in the amount of procaspase-3 detected by western blot (Figure 5A, left hand panel). When HDMEC cultured on plastic were treated with various doses of IP-10 (Figure 5B, left hand panel) or endostatin (Figure 5C, left hand panel) alone or in combination with VEGF, we observed essentially no change in the levels of procaspase-3 suggesting that there is no active caspase-3 produced following HDMEC stimulation with these inhibitors. We observed no change in procaspase-3 levels when HDMEC were cultured on collagen I and stimulated with TSP-1 alone as compared to unstimulated controls (Figure 5A, right hand panel, lanes 3, 5 and 7 as compared to lane 1). We did however observe decreases in procaspase-3 with increasing concentrations of TSP-1 when HDMEC grown on collagen I were treated with TSP-1 in combination with VEGF, indicating that at higher doses of TSP-1, activation of caspase-3 following procaspase-3 cleavage occurs even in the presence of collagen I (Figure 5A, right hand panel, lanes 4, 6 and 8 as compared to lane 2). It would appear that under certain conditions, VEGF prevents the cleavage of procaspase-3 into active caspase-3 to some extent, however we did not consistently observe this phenomenon. It should also be noted that unstimulated HDMEC on plastic or collagen I have a fairly high apoptotic rate with approximately 40–50% of unstimulated cells being apoptotic after 62 h of culture (data not shown). Therefore, the baseline level of procapase-3 seen in the unstimulated condition inherently reflects a certain level of caspase-3 activation. Even though we detected a decrease in procaspase-3 indicating that caspase-3 had been activated and proteolytically processed following treatment of HDMEC cultured on collagen I with TSP-1 and VEGF as compared to treatment with VEGF alone (Figure 5A, right hand panel), we surprisingly did not observe significant increases in the apoptosis of TSP-1 and VEGF treated cells on collagen I. It is possible that the degree of procaspase-3 cleavage was insufficient to induce significant apoptosis in this system or that growth on collagen I induces a block in the apoptotic pathway downstream of caspase-3. Alternatively, it is possible that measurement of the reduction in procaspase-3 levels is not indicative of the generation of active caspase-3. In contrast to TSP-1, we saw very little changes in the levels of procaspase-3 following stimulation of HDMEC on collagen I with either IP-10 (Figure 5B, right hand panel) or endostatin (Figure 5C, right hand panel) alone or in combination with VEGF.

**Figure 6:**
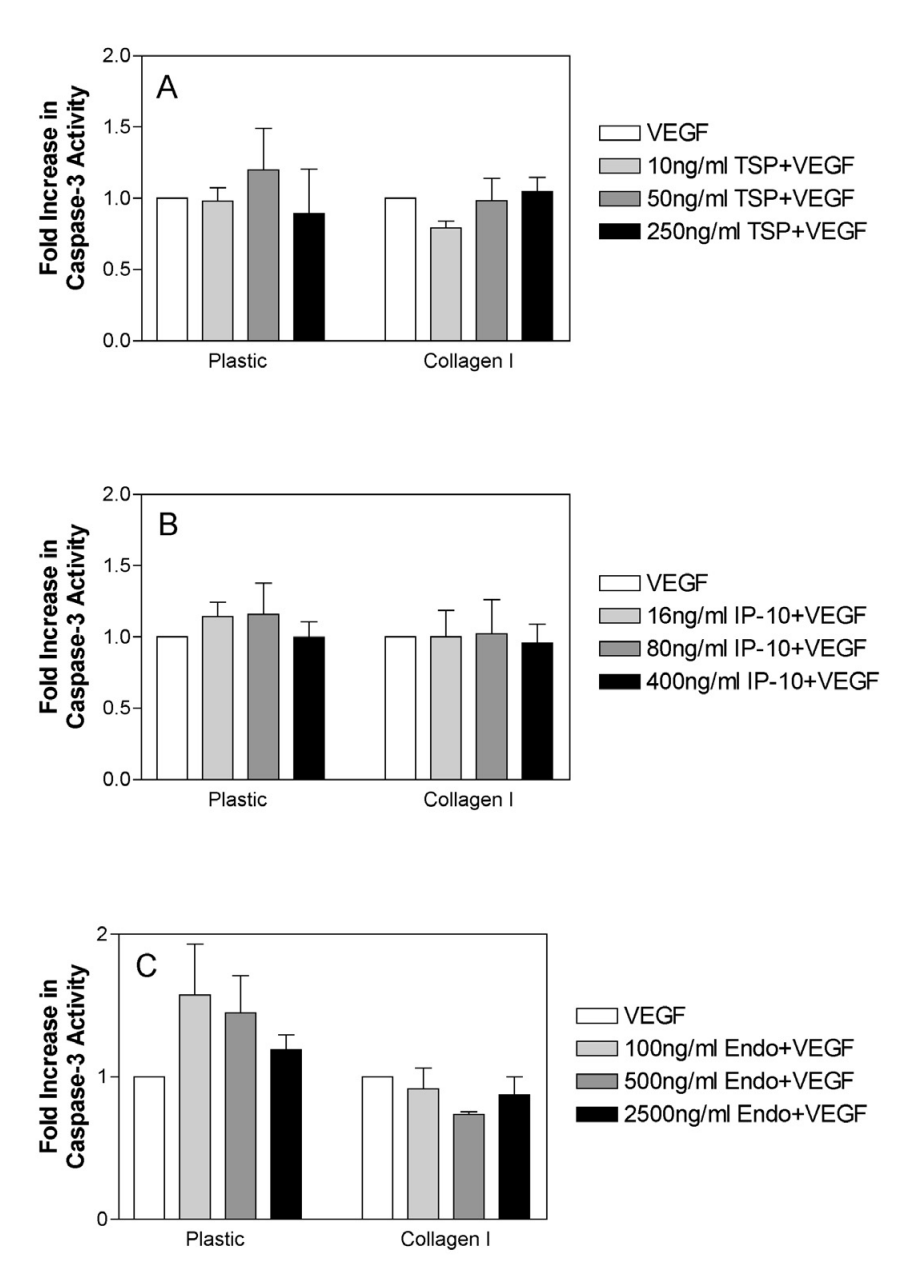
**Activation of caspase-3 is abrogated by VEGF stimulation in HDMEC cultured on collagen I**. HDMEC were seeded on either plastic or collagen I coated tissue culture dishes, and following removal of non-adherent cells by washing, were stimulated with either 50 ng/ml VEGF alone or in combination with 10, 50 or 250 ng/ml TSP-1 (panel A), 16, 80 or 400 ng/ml IP-10 (panel B), or 100, 500 or 2500 ng/ml recombinant endostatin (panel C) for 48 h. Cells were collected following collagenase treatment and counted. Cell pellets were lysed in appropriate volumes of lysis buffer to generate extracts containing equal number of cells per unit volume. A fluorometric based assay for caspase-3 activity was performed using Ac-DEVD-AMC as a substrate as described in the methods section. Bars are representative of the mean and standard error for triplicate samples. The experiment was performed three independent times with similar results. Statistical significance was determined using unpaired t-tests as compared to unstimulated samples or to VEGF stimulated samples for TSP-1, IP-10 or endostatin alone or in combination with VEGF respectively (* = p < 0.05).

As decreases in procaspase-3 levels may not necessarily be reflective of caspase-3 activation, we performed additional experiments to detect active caspase-3 using a fluorometric assay based on the cleavage of the caspase-3 substrate Ac-DEVD-AMC. As with previous experiments, cells were cultured on plastic or collagen I and stimulated with the indicated doses of TSP-1, IP-10 or endostatin in the presence of 50 ng/ml VEGF. Although none of the conditions reached statistical significance, we observed a trend for increased caspase-3 activity in HDMEC treated with TSP-1 (Figure 6A), IP-10 (Figure 6B), or endostatin (Figure 6C) in combination with VEGF following culture on plastic. We did not observe these same increases in caspase-3 activity following treatment of HDMEC with TSP-1, IP-10 or endostatin in combination with VEGF when cells were cultured on collagen I (Fig. 6). These results support the results obtained by western blot analysis and suggest that when HDMEC are cultured on collagen I activation of caspase-3 is inhibited and cells do not undergo apoptosis in response to inhibitors as a result.

**Figure 7:**
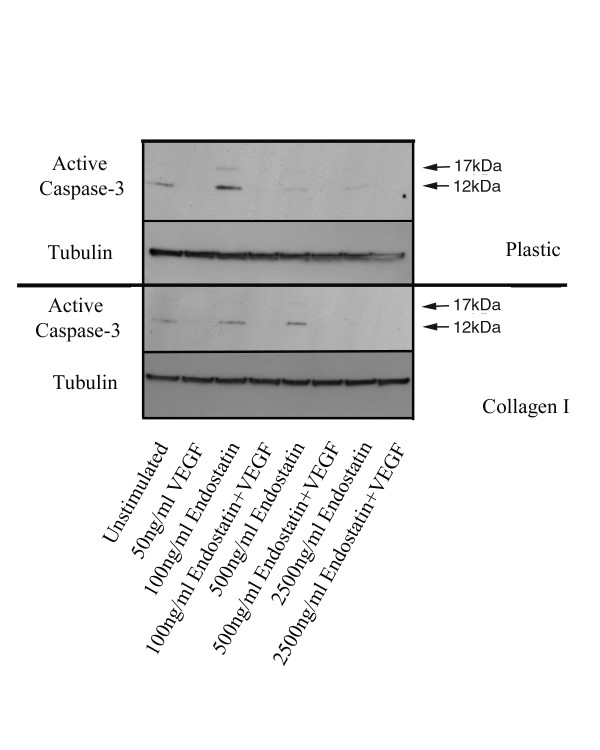
**Endostatin does not induce cleavage of caspase-3 into its active forms**. HDMEC were seeded on either plastic or collagen I coated tissue culture dishes, and following removal of non-adherent cells by washing, were stimulated with either 50 ng/ml VEGF alone or in combination with 100, 500 or 2500 ng/ml recombinant endostatin for 48 h. Cells were collected by trypsinization or collagenase treatment and total protein lysates were generated. Western blots for active caspase-3 were performed as described in the methods section.

Since we observed the induction of apoptosis of HDMEC following treatment by endostatin when cells were cultured on collagen I, we were surprised to find that caspase-3 did not appear to be activated by endostatin. As both detection of procaspase forms and the fluorometric activity assays are known to be variable, we performed western blots using an antibody to the active form of caspase-3, which readily detects the 17 and 12 kDa cleavage products, in an attempt to confirm our previous findings. We found that when cultured on plastic, a slight increase in the active form of caspase-3 could be observed following treatment with 100 ng/ml of endostatin alone as compared to unstimulated cells (Fig. 7, top panel, lane 3 as compared to lane 1). However, in the presence of VEGF, there was no detectable activation of caspase-3 at any of the doses of endostatin tested (Fig. 7, top panel, lanes 4, 6 and 8 as compared to lane 2). Similarly, although some active caspase-3 could be detected following endostatin treatment of HDMEC cultured on collagen I, this activity was not increased as compared to that observed in the unstimulated HDMEC (Fig. 7, bottom panel, lanes 3, 5 and 7 as compared to lane 1). In support of our previous data, we observed no increases in the levels of the active forms of caspase-3 following stimulation of HDMEC on collagen I with VEGF in combination with endostatin at any of the doses tested (Fig. 7, bottom panel, lanes 4, 6 and 8 as compared to lane 2). The lack of active caspase-3 cleavage products following treatment with VEGF also supports the contention that VEGF can increase the survival potential of endothelial cells by reducing their apoptotic rate. Taken together, our data suggests that endostatin is the sole angiostatic molecule tested in this system that retains the ability to induce apoptosis of endothelial cells following growth on collagen I. Surprisingly however, the endostatin-induced apoptosis appears to occur via a caspase-3 independent mechanism that is insensitive to signals mediated through attachment of cells to collagen I.

### Endostatin induces the activation of caspase-8 in HDMEC grown on collagen I

**Figure 8:**
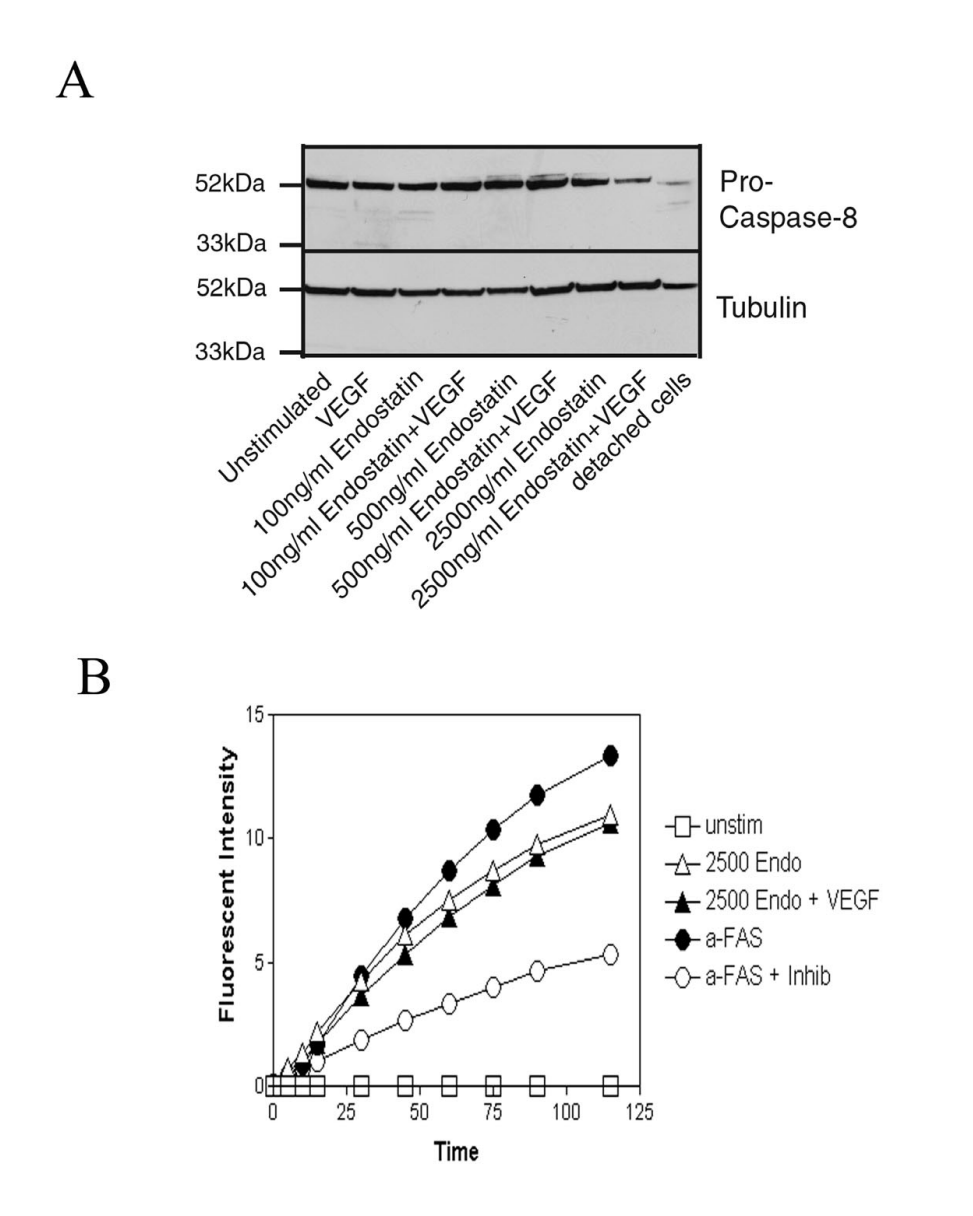
**Endostatin induces the activity of caspase-8**. **A) **HDMEC were seeded on tissue culture dishes, and following removal of non-adherent cells by washing, were stimulated with either 50 ng/ml VEGF alone or in combination with 100, 500 or 2500 ng/ml recombinant endostatin for 48 h. Cells were collected and total protein lysates were generated. Western blots for procaspase-8 were performed as described in materials and methods. Blots were stripped and reprobed for tubulin as a loading control. **B) **HDMEC were seeded on tissue culture dishes, and following removal of non-adherent cells by washing, were stimulated with either regular media (unstimulated), or media containing 100, 500, or 2500 ng/ml endostatin, in the presence or absence of 50 ng/ml VEGF, or stimulated with anti-fas antibody as a positive control for caspase-8 activation. Cells were collected by trypsinization and cell pellets stored at -80°C. Subsequently, pellets were lysed and total protein concentration was determined. Caspase-8 activity was then detected using a commercially available fluorometric detection kit (Sigma, Oakville, ON) as described in the methods section. An additional sample was included using the anti-fas treated cell lysate in the presence of specific caspase-8 inhibitors to demonstrate the specificity of the assay.

It has been demonstrated that apoptosis of cells may occur through activation of "death receptors" that primarily mediate their effects via activation of caspase-8 (recently reviewed by [38]). As caspase-8 can also be activated by other mechanisms independent of death receptor activation [39,40], we sought to determine whether the mechanism of endostatin-induced apoptosis of endothelial cells was caspase-8 dependent. We thus examined the expression of caspase-8 following treatment of cells with various doses of endostatin alone or in combination with 50 ng/ml VEGF. We could detect a reduction in procaspase-8, suggesting cleavage into its active forms in HDMEC treated with 2500 ng/ml of endostatin alone or in combination with 50 ng/ml VEGF (Fig. 8A, lane 7 as compared to lane 1, and lane 8 as compared to lane 2). Endothelial cells that were not allowed to adhere to substratum and are known to undergo apoptosis via a fas-mediated mechanism in this situation were used as a positive control for procaspase-8 cleavage and activation (Fig. 8A, lane 9). We further confirmed these results following the detection of active caspase-8 using a fluorometric assay based on cleavage of the substrate Ac-IETD-AMC. HDMEC were stimulated with the various concentrations of endostatin alone or in combination with VEGF as indicated. As a positive control, an activating antibody to fas receptor was added to cultured cell supernatants to induce cleavage of caspase-8 (Fig. 8B, closed diamonds). To demonstrate the specificity of the activity in the assay, cells were also treated with anti-fas antibody in the presence of a specific caspase-8 inhibitor (Fig. 8B, open diamonds). Active caspase-8 could only be detected in HDMEC treated with 2500 ng/ml of endostatin alone or in combination with 50 ng/ml VEGF (Fig. 8B, open and closed upright triangles respectively). We did not detect active caspase-8 following stimulation with the lower doses of endostatin either by western blot or activity assays. These data suggest that endostatin mediates endothelial cell apoptosis in part by the activation of caspase-8, at least at higher doses. It is possible that endostatin induces apoptosis at the lower doses tested by activating caspase-8 at a level that is below our threshold of detection, or that at lower doses endostatin induces apoptosis via a caspase-8 independent pathway.

### Endostatin but not TSP-1 or IP-10 inhibits VEGF-induced HDMEC sprout-formation on collagen I gels

**Figure 9:**
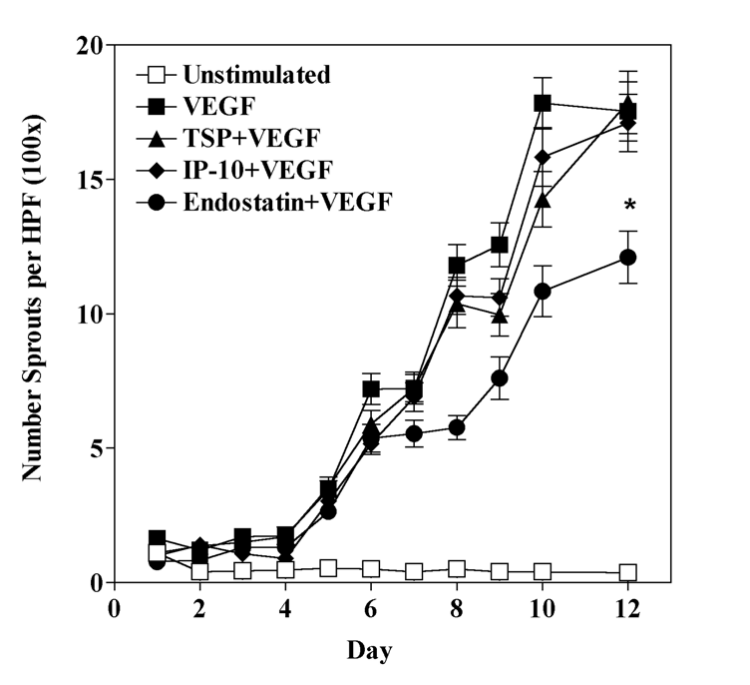
**Endostatin, but not TSP-1 or IP-10, can inhibit VEGF-induced tube-formation on collagen I gels**. HDMEC were seeded on collagen I gels, and following removal of non-adherent cells by washing, were stimulated with media alone (unstimulated) or media containing 50 ng/ml VEGF alone or in combination with 50 ng/ml TSP-1, 80 ng/ml IP-10 or 500 ng/ml endostatin. Cells were counted daily and media was replenished every second day. Graph is representative of the mean and standard error of the number of sprouts counted in 12 random fields of view at 100× magnification in triplicate dishes (n = 24). Statistical significance was determined at day 12 using unpaired t-tests as compared to VEGF treated cells (* = p < 0.05).

An additional manner by which angiogenesis might be inhibited is via the prevention of the organization of endothelial cells into vessel-like structures. To determine the ability of TSP, IP-10 and endostatin to inhibit this process we utilized an *in vitro *tube-formation assay that uses collagen I gels as a substrate. In this system, HDMEC seeded on fibrillar collagen I gels do not spontaneously form tube-like structures, but only do so after stimulation with angiogenic factors such as VEGF. Cells will initially align with one another and begin to extend themselves into an initial "sprouting" cell; and then following 8–12 days in culture, they will form multicellular tube structures with lumen. HDMEC were seeded on collagen I gels and were then stimulated with 50 ng/ml VEGF alone or in combination with either 50 ng/ml TSP-1, 80 ng/ml IP-10 or 500 ng/ml endostatin. The number of multicellular tube-like structures was counted daily for a period of 12 days. We found that neither TSP-1 nor IP-10 could inhibit the VEGF-induced tube formation in this assay system (Fig. 9, triangles and diamonds respectively). In contrast, we observed that endostatin significantly inhibited VEGF-induced tube formation on collagen I (Fig. 9, circles). Interestingly, it would appear that endostatin did not interfere with the early stages of tube formation and cells were able to align with one another, however their ability to organize into tube structures with lumen was significantly impaired. These data again support the notion that the mechanisms by which TSP-1 and IP-10 inhibit endothelial cells and angiogenesis are different than that of endostatin. Furthermore, it would appear that the mechanisms of inhibition induced by TSP-1 and IP-10 are affected by growth of HDMEC on collagen I while endostatin retains its ability to inhibit endothelial cell survival and organization in the presence of signals induced by growth of cells on collagen I.

## Discussion

We had previously observed that VEGF enhanced survival of HDMEC grown on collagen I [31], suggesting that factors such as elevated collagen I levels within tumor microenvironments could increase endothelial cell survival and thus could potentially induce resistance to angiostatic molecules *in vivo*. We decided to formally examine this hypothesis and determined that growth on the tumor-associated matrices collagen I and tenascin C impaired the ability of TSP-1 and IP-10 to inhibit endothelial cell proliferation and induce endothelial cell apoptosis (in the case of collagen I). The ability of TSP-1 to inhibit endothelial cell proliferation has previously been suggested to be dependent on its ability to bind to heparin and compete with heparin-binding growth factors for binding sites on cells [41]. As TSP-1 has been previously shown to be able to bind collagen I, it is quite possible that TSP-1 is sequestered from cells by binding collagen I, thus allowing the heparin-binding growth factors to stimulate the proliferation of HDMEC on collagen I [42]. Thrombospondin has also been previously shown to induce apoptosis in endothelial cells via activation of caspase-3 [8,9]. Surprisingly, although we did see a dose-dependent decrease in the levels of procaspase-3 following treatment of HDMEC on collagen I with TSP-1 in combination with VEGF, indicating activation of caspase-3, this was not associated with the induction of significant apoptosis as compared to stimulation with VEGF alone. Unlike other studies suggesting that TSP-1 induced endothelial cell apoptosis in a caspase-3 dependent manner [8,9], our studies were performed in the presence of exogenous VEGF, thus although some processing of caspase-3 occurs in response to TSP-1 in our experimental conditions, it is not enough to overcome the survival effects induced by simultaneous exogenous VEGF stimulation. Other studies have implicated caspase-8, fas and fasL as the main mediators of TSP-1-induced apoptosis [43]. We attempted to determine if caspase-8 was playing a role in TSP-1-induced apoptosis in our model system, however were unable to detect active caspase-8 using western blot assays. There are a number of differences between our study and those previously reported, including different endothelial cell types that are known to behave differently in experimental conditions [44-46] and different TSP-1 concentrations than those used in our studies. In fact, it was reported that when TSP-1 was delivered in combination with 50 ng/ml of VEGF, the cells became resistant to TSP-1 [43], thus confirming our observations.

We also observed that growth on collagen I prevented the angiostatic chemokine IP-10 from inhibiting endothelial cell proliferation and inducing endothelial cell apoptosis. The mechanisms of endothelial cell inhibition by IP-10 have not been extensively studied. Previous data has suggested that IP-10 can inhibit endothelial cell proliferation [47], however other reports suggest that IP-10 had no effect on endothelial cell growth, attachment or migration, but did however inhibit bFGF-induced tube formation *in vitro *and *in vivo *[18]. More recently, using IP-10 mutants that have impaired binding domains, the angiostatic activity of IP-10 was shown to be dependent on its binding to the g-protein coupled receptor CXCR3 [48]. It has been previously demonstrated that cell migration in response to SDF-1 via activation of CXCR4 is enhanced in the presence of fibronectin, suggesting that signaling through integrins can affect the downstream signal transduction pathway activated by the CXC receptors [49]. To date, there are no studies implicating integrins in CXCR3 signaling, however the CXCR family of receptors are very homologous so it is highly possible that ECM binding through the integrins can modulate the response following CXCR3 activation. As very little is known about the mechanism by which IP-10 inhibits angiogenesis, further investigation into the mechanism of inhibition of endothelial cell proliferation and induction of apoptosis by IP-10, and the effects of tumor-associated extracellular matrices is warranted.

We also examined the ability of endostatin to inhibit endothelial cell proliferation following growth on plastic, collagen I, laminin or tenascin C. As seen with the other inhibitors, endostatin was not able to inhibit endothelial cell proliferation when HDMEC were cultured on collagen I or tenascin C as a substrate. Previous data suggested that inhibition of proliferation of endothelial cells is one of the primary mechanisms of its anti-angiogenic capabilities [22,24]. Subsequently, endostatin has been shown to affect multiple facets of the angiogenic process including cell migration [23,24], survival [50], protease activity [51,52], and vessel stabilization [50,53]. Discrepancies in the results obtained using endostatin can be observed depending on the source of endostatin used in the experiments, which can be either bacterially derived [24], purified from murine hemangioendothelioma cells [22], or derived from Pichia pastoris. We have used the latter source of endostatin for our studies, and it has previously been shown to inhibit endothelial cell proliferation and migration and to induce a G1 arrest in cells [54]. Our observations support previous observations that endostatin can inhibit VEGF-induced endothelial cell proliferation to some extent; however, we have extended these findings to demonstrate that in the presence of the "tumor-associated" ECM proteins collagen I and tenascin C, this inhibition of proliferation by endostatin is dramatically impaired. The receptor that binds endostatin to inhibit angiogenic activities remains unclear despite numerous studies indicating its ability to bind cell surface molecules such as glypicans [55], heparin [56], tropomyosin-3 [57], VEGFR1 and VEGFR2 [58], and integrins α_v _and α_5 _[59]. It has been previously shown that α_5_β_1 _integrins cluster following endostatin binding [60], and since collagen I and tenascin C can both bind β_1 _integrins (in association with various α integrin chains) it is highly possible that binding of these matrices by integrins could modulate either the affinity for endostatin binding or integrin signal transduction cascades through an inside-out signal [61]. Furthermore, a peptide derived from endostatin has recently been shown to be a potent inhibitor of angiogenesis in a β_1 _integrin and heparin-dependent manner [62], suggesting that perhaps collagen I and tenascin C compete with endostatin for integrin-binding on cells and supporting our observations that endostatin was ineffective at inhibiting endothelial cell proliferation following growth on collagen I or tenascin C.

In contrast to our results with endothelial cell proliferation, we found that endostatin could induce apoptosis of endothelial cells regardless of whether they were cultured on plastic or collagen I substrates. In fact, we observed endothelial cell apoptosis at doses 100-fold lower than has previously been described [25]. The previous studies indicated that endostatin's ability to induce apoptosis was due to its ability to decrease bcl-2 expression and induce activation of caspase-3 [25]. In contrast to these results, we did not observe either a decrease in bcl-2 expression nor activation of caspase-3 in HDMEC following endostatin stimulation in either the presence or absence of VEGF. In these studies, bovine pulmonary artery endothelial cells were used, thus differences observed between the doses of endostatin required to induce apoptosis could again be a reflection of differences in the behavior of different endothelial cell types.

As endostatin has been previously shown to bind to a number of different integrin molecules [59], and recently it has been demonstrated that the inappropriate ligation of integrins can lead to what has been referred to as integrin-mediated death [63], it is possible that endostatin may induce apoptosis via alternative mechanisms such as integrin-mediated death. This form of apoptosis was shown to be dependent on caspase-8, and our observation of activation of caspase-8 observed at the higher doses of endostatin supports the notion that the apoptosis observed in our system following treatment with endostatin may be related to integrin-mediated death. Recently, it has also been shown that endostatin inhibited the migration on and attachment of endothelial cells to collagen I, but did not affect the proliferation of endothelial cells cultured on this matrix [64], which is in complete agreement with our results. Other evidence would also suggest that the main mechanism of inhibition of angiogenesis by endostatin is via its ability to inhibit cell migration through α_5_β_1 _integrin, heparan sulfate, and lipid raft-mediated interactions [65], supporting our observations regarding endostatin's ability to inhibit vessel formation in our tube formation assays, where endothelial cell migration is a prerequisite for structure formation, and our results would suggest that this effect is independent of signals from collagen I.

In conclusion, we have demonstrated that TSP, IP-10 and endostatin mediate inhibition of angiogenesis via different mechanisms that are affected to different degrees by growth of the endothelial cells on substrates such as collagen I. Our data suggests that the biological effects that various angiostatic molecules have on endothelial cells may be affected by the type of extracellular matrix upon which the cells are in contact. Indeed, the importance of cues from the extracellular matrix in vessel survival are also evident from transgenic animal systems where targeted deletion of the collagen I gene in mice led to embryonic death, due to rupture of blood vessels [66]. The effects of ECM on vessel survival and angiogenesis is extremely important in the context of inhibition of angiogenesis as an anti-tumor therapy as tumors usually have extensively remodeled matrix with differences in the composition of this matrix as compared to that found in normal tissues. Given the differences in the human tumor matrix microenvironment, and our results that "tumor-associated" matrices such as collagen I and tenascin C may enhance the resistance of endothelial cells to certain anti-angiogenic agents, it is imperative that we gain a clearer understanding of the mechanism of inhibition of angiogenesis by these molecules and how this may be affected by differences in the tumor microenvironment. These insights will potentially help to predict patient response to these inhibitors and elucidate targets of intervention that will be unaffected by differences in the tumor matrix microenvironment.

## Methods

### Endothelial cells and antibody reagents

Human dermal microvascular endothelial cells (HDMEC) derived from neonatal tissue were obtained from Cambrex Corporation (Walkersville, MD) and were propagated in EGM-2MV media (Cambrex Corp., Walkersville, MD). All experiments were performed using cells between passages 4 and 10. Rabbit anti-Bax and goat anti-Caspase-3 antibodies were from R&D Systems (Minneapolis, MN). Hamster anti-Bcl-2 antibody was purchased from BD Pharmingen (Mississauga, ON) and rabbit anti-active caspase-3 antibody was from Biovision (Palo Alto, CA). Mouse anti-caspase-8 antibody was purchased Cell Signaling Technologies (Beverly, MA).

### Coating tissue culture dishes with collagen

Vitrogen 100 bovine dermal collagen (Cohesion Technologies, Palo Alto, CA) was used to coat tissue culture vessels in all experiments, and is a mixture of ~97% collagen I and ~3% collagen III matrices. The acidified collagen solution was kept on ice, diluted to a concentration of 1.5 mg/ml, and neutralized following addition of 10× PBS and 0.1 N NaOH to a pH of approximately 7.4. The appropriate volume of collagen solution was added to coat each vessel and the plates were rocked to ensure even distribution of collagen across the surface. Plates were then incubated at 37°C for 4 h to overnight to allow gelation to occur. The collagen surfaces were washed with Hanks Buffered Salt Solution (HBSS, Invitrogen, Carlsbad, CA), and incubated in EGM-2MV for a minimum of 2 h to equilibrate the collagen prior to addition of endothelial cells. For coating with laminin, human placenta laminin (Sigma, Oakville, ON) was diluted in PBS and added to dishes at a concentration of 1 μg/cm^2^. The dishes were allowed to dry overnight, uncovered, in a laminar-flow hood and cells were seeded on the surfaces the next day. For tenascin C, dishes were coated with tenascin C purified from a human tumor cell line (Chemicon International, Temecula, CA). For coating, tenascin-C was diluted to 0.1 μg/ml in PBS, and added to dishes which were then incubated overnight at 4°C. The next day, the solution was aspirated, and the dishes were blocked with 0.1% casein solution for at least 1 h. The dishes were then washed three times with 1× PBS, and seeded with cells.

### Proliferation assays

HDMEC were seeded at a density of 15,000 cells per well in EGM-2MV into untreated or matrix coated 12-well plates as described above and allowed to incubate overnight. The next day, media was removed, cells were washed twice with HBSS and then starved overnight in MCDB 131 (Invitrogen, Carlsbad, CA) containing 1% fetal bovine serum (FBS). The following day cells were stimulated with varying concentrations of either TSP-1 (Calbiochem, San Diego, CA), IP-10 (Intergen, Purchase, NY), or endostatin (Calbiochem, San Diego, CA) together with 50 ng/ml VEGF (kind gift from the Biological Resources Branch of the National Cancer Institute) in MCDB 131 containing 5% FBS. Stimulation with 50 ng/ml VEGF in MCDB 131 containing 5% FBS was used as a positive control for proliferation, and stimulation with MCDB 131 containing only 1% FBS was used as a negative control for proliferation. Following 72 h of incubation, cells were collected by trypsinization or by collagenase treatment in the case of cells seeded on collagen substrate, and the number of cells per unit volume in each well was determined following counting in a Coulter counter. Each treatment was performed in triplicate and the entire experiment was performed a minimum of three independent times.

### BrdU Cell Proliferation ELISA

HDMEC were seeded at a density of 2,500 cells per well in EGM-2MV into untreated or collagen I coated 96-well plates as described above and allowed to incubate overnight. The next day, media was removed, cells were washed twice with HBSS and then starved overnight in MCDB 131 containing 1% FBS. The following day cells were stimulated with varying concentrations of either TSP-1, IP-10, or endostatin together with 50 ng/ml VEGF in MCDB 131 containing 5% FBS. BrdU labeling solution was subsequently added 48 h post-stimulation with growth factors and inhibitors, and cells were incubated overnight. Incorporated BrdU was subsequently detected using the BrdU Cell Proliferation ELISA, kit according to the manufacturers directions (Roche, Laval, PQ), using a Fluoroskan Ascent Plate reader (Thermolabs, Franklin, MA) for luminescent detection.

### Determination of apoptosis by FACS

HDMEC were seeded into untreated or collagen I coated dishes and allowed to adhere overnight. The following day, monolayers were washed with HBSS to remove non-adherent cells, and adherent cells were stimulated with MCDB 131 media containing 5% FBS (unstimulated), or MCDB 131 media with 5% FBS supplemented with 50 ng/ml VEGF alone, 10, 50 or 250 ng/ml TSP-1, 16, 80 or 400 ng/ml IP-10, or 100, 500 or 2500 ng/ml endostatin alone or in combination with 50 ng/ml VEGF. Cells were incubated for 60 h, and then harvested by collecting the non-adherent and adherent cell populations either by trypsinization or collagenase treatment for plastic and collagen I coated dishes respectively. Collected cells were pelleted by centrifugation at 300 × g, washed twice with PBS, and then resuspended in ice-cold 70% ethanol. Cell suspensions were incubated in 70% ethanol at -20°C for a minimum of 24 h. Following permeabilization, cells were washed two times with PBS, and then resuspended in 500 ul of propidium iodide solution (48 ug/ml propidium iodide, 40 ug/ml Rnase A in PBS). The percentage of apoptotic cells was determined following identification of the sub-G1 population of cells by flow cytometric analysis.

### Detection of caspase-3 or caspase-8 activity

HDMEC were seeded into untreated or collagen I coated dishes and allowed to adhere overnight. The following day, monolayers were washed with HBSS to remove non-adherent cells, and cells were stimulated with EGM-2MV media alone, 50 ng/ml VEGF alone, or 50 ng/ml TSP-1, 80 ng/ml IP-10, or 500 ng/ml endostatin alone or in combination with 50 ng/ml VEGF in EGM-2MV. Cells were incubated for 48 h, and then harvested by collecting the non-adherent and adherent cell population by either trypsinization or collagenase treatment for plastic and collagen I coated dishes respectively. Collected cells were pelleted by centrifugation at 300 × g, washed with PBS, counted and then stored at -80°C. At time of assay, cells were lysed in the appropriate volume of lysis buffer (10 mM HEPES, 1 mM EDTA, 100 mM NaCl, 5 mM MgCl, 142.5 mM KCl, and 1 mM DTT) so that equal cell number/unit volume was obtained for each sample. Samples were incubated on ice for 45 minutes followed by a centrifugation at 13,000 rpm at 4°C for 30 minutes in a microfuge. Supernatants were removed and 20 ul of each sample was added to assay buffer (50 mM HEPES, 1 mM EDTA, 100 mM NaCl, 10% glycerol, 0.1% CHAPS, 10 mM DTT, pH 7.4) in a 96-well microtiter plate. Plates were allowed to equilibrate at 37°C for 10 minutes, and then 10 ul of a 1 mM stock of the fluorogenic caspase-3 substrate Ac-DEVD-AMC (Alexis Biochemicals, San Diego, CA) was added to each well. Plates were incubated at 37°C to allow the enzymatic reaction to proceed, and the fluorescence was measured at various times post-addition of substrate using a fluorescent plate reader set at 460 nm emission/360 nm excitation wavelengths. Purified caspase-3 was used as a positive control in all assays, and all samples were assayed in triplicate. Experiments were performed a minimum of three independent times.

For detection of caspase-8 activity, cells were seeded into dishes and stimulated as described above. Following incubation for 48 h, cells were isolated following trypsinization, washed once with PBS, and cell pellets were stored at -80°C. Subsequently, the pellets were lysed in provided caspase activity lysis buffer according to the manufacturer's directions (Sigma, Oakville, ON), the protein concentration for each lysate was determined, and caspase-8 activity was determined according to the manufacturer, using a fluorogenic caspase-8 activity kit (Sigma, Oakville, ON) that used Ac-IETD-AMC as a substrate and fluorometric detection in a fluorescent plate reader set at 460 nm emission/360 nm excitation wavelengths.

### Western blot analysis

HDMEC were seeded on plastic or collagen I coated dishes as described above and allowed to adhere overnight. The next day, monolayers were washed twice with HBSS and then stimulated with varying concentrations of TSP-1, IP-10 or endostatin alone or in combination with 50 ng/ml VEGF. Total protein lysates were generated in boiling lysis buffer (1% SDS, 1.0 mM sodium ortho-vanadate, 10 mM Tris pH 7.4, 0.2 mM PMSF, 2 μg/ml aprotinin) following recovery of cells from dishes by trypsinization or collagenase treatment for growth on plastic or collagen I respectively. Aliquots containing 5–40 μg of total protein were subjected to SDS-PAGE electrophoresis followed by transfer to nitrocellulose membranes. Specific proteins were detected following incubation with primary and horse-radish peroxidase-conjugated secondary antibodies and visualization using chemiluminescent detection (Supersignal, Pierce, Rockford, MD).

### Endothelial cell sprouting assays

Collagen I gels were prepared as described above. 2 × 10^5 ^HDMEC were seeded onto each 60 mm dish of collagen I and allowed to adhere overnight. The following day the dishes were washed twice with HBSS and then stimulated with EGM-2MV alone or supplemented with 50 ng/ml VEGF alone or in combination with 50 ng/ml TSP, 80 ng/ml IP-10 or 500 ng/ml endostatin. Cells were counted on day 0 prior to stimulation to ensure similar numbers of cells had been seeded on each dish, and were then counted daily for 12 days. Media containing supplements was replaced every 48 hours.

## Authors' contributions

CLA contributed to the conception, design, acquisition and analysis of data in addition to writing the manuscript and performing the BrdU Cell proliferation ELISA and tube-formation assays. JEN contributed intellectually to the study and critical evaluation of the manuscript. SAL performed the caspase-3 activity assays and contributed to the critical evaluation of the manuscript. PJP contributed intellectually to the study and critical evaluation of the manuscript. HZ performed western blot analyses and CED performed western blot analyses, caspase-8 activity assays, and apoptosis experiments in addition to contributing to the critical evaluation of the manuscript.

## References

[B1] Gimbrone MAJ, Leapman SB, Cotran RS, Folkman J (1972). Tumor dormancy in vivo by prevention of neovascularization. J Exp Med.

[B2] Baenziger NL, Brodie GN, Majerus PW (1972). Isolation and properties of a thrombin-sensitive protein of human platelets. J Biol Chem.

[B3] Dixit VM, Haverstick DM, O'Rourke KM, Hennessy SW, Grant GA, Santoro SA, Frazier WA (1985). A monoclonal antibody against human thrombospondin inhibits platelet aggregation. Proc Natl Acad Sci U S A.

[B4] Leung LL (1984). Role of thrombospondin in platelet aggregation. J Clin Invest.

[B5] O'Shea KS, Dixit VM (1988). Unique distribution of the extracellular matrix component thrombospondin in the developing mouse embryo. J Cell Biol.

[B6] Taraboletti G, Roberts D, Liotta LA, Giavazzi R (1990). Platelet thrombospondin modulates endothelial cell adhesion, motility, and growth: a potential angiogenesis regulatory factor. J Cell Biol.

[B7] Guo N, Krutzsch HC, Inman JK, Roberts DD (1997). Thrombospondin 1 and type I repeat peptides of thrombospondin 1 specifically induce apoptosis of endothelial cells. Cancer Res.

[B8] Jimenez B, Volpert OV, Crawford SE, Febbraio M, Silverstein RL, Bouck N (2000). Signals leading to apoptosis-dependent inhibition of neovascularization by thrombospondin-1. Nat Med.

[B9] Nor JE, Mitra RS, Sutorik MM, Mooney DJ, Castle VP, Polverini PJ (2000). Thrombospondin-1 induces endothelial cell apoptosis and inhibits angiogenesis by activating the caspase death pathway. J Vasc Res.

[B10] Castle V, Varani J, Fligiel S, Prochownik EV, Dixit V (1991). Antisense-mediated reduction in thrombospondin reverses the malignant phenotype of a human squamous carcinoma. J Clin Invest.

[B11] Castle VP, Dixit VM, Polverini PJ (1997). Thrombospondin-1 suppresses tumorigenesis and angiogenesis in serum- and anchorage-independent NIH 3T3 cells. Lab Invest.

[B12] Rastinejad F, Polverini PJ, Bouck NP (1989). Regulation of the activity of a new inhibitor of angiogenesis by a cancer suppressor gene. Cell.

[B13] Sheibani N, Frazier WA (1995). Thrombospondin 1 expression in transformed endothelial cells restores a normal phenotype and suppresses their tumorigenesis. Proc Natl Acad Sci U S A.

[B14] Luster AD, Ravetch JV (1987). Biochemical characterization of a gamma interferon-inducible cytokine (IP-10). J Exp Med.

[B15] Sarris AH, Esgleyes-Ribot T, Crow M, Broxmeyer HE, Karasavvas N, Pugh W, Grossman D, Deisseroth A, Duvic M (1995). Cytokine loops involving interferon-gamma and IP-10, a cytokine chemotactic for CD4+ lymphocytes: an explanation for the epidermotropism of cutaneous T-cell lymphoma? [see comments]. Blood.

[B16] Taub DD, Longo DL, Murphy WJ (1996). Human interferon-inducible protein-10 induces mononuclear cell infiltration in mice and promotes the migration of human T lymphocytes into the peripheral tissues and human peripheral blood lymphocytes-SCID mice. Blood.

[B17] Strieter RM, Kunkel SL, Arenberg DA, Burdick MD, Polverini PJ (1995). Interferon gamma-inducible protein 10 (IP-10), a member of the C-X-C chemokine family, is an inhibitor of angiogenesis. Biochem Biophys Res Commun.

[B18] Angiolillo AL, Sgadari C, Taub DD, Liao F, Farber JM, Maheshwari S, Kleinman HK, Reaman GH, Tosato G (1995). Human interferon-inducible protein 10 is a potent inhibitor of angiogenesis in vivo. J Exp Med.

[B19] Arenberg DA, Kunkel SL, Polverini PJ, Morris SB, Burdick MD, Glass MC, Taub DT, Iannettoni MD, Whyte RI, Strieter RM (1996). Interferon-gamma-inducible protein 10 (IP-10) is an angiostatic factor that inhibits human non-small cell lung cancer (NSCLC) tumorigenesis and spontaneous metastases. J Exp Med.

[B20] Feldman AL, Friedl J, Lans TE, Libutti SK, Lorang D, Miller MS, Turner EM, Hewitt SM, Alexander HR (2002). Retroviral gene transfer of interferon-inducible protein 10 inhibits growth of human melanoma xenografts. Int J Cancer.

[B21] Regulier E, Paul S, Marigliano M, Kintz J, Poitevin Y, Ledoux C, Roecklin D, Cauet G, Calenda V, Homann HE (2001). Adenovirus-mediated delivery of antiangiogenic genes as an antitumor approach. Cancer Gene Ther.

[B22] O'Reilly MS, Boehm T, Shing Y, Fukai N, Vasios G, Lane WS, Flynn E, Birkhead JR, Olsen BR, Folkman J (1997). Endostatin: an endogenous inhibitor of angiogenesis and tumor growth. Cell.

[B23] Yamaguchi N, Anand-Apte B, Lee M, Sasaki T, Fukai N, Shapiro R, Que I, Lowik C, Timpl R, Olsen BR (1999). Endostatin inhibits VEGF-induced endothelial cell migration and tumor growth independently of zinc binding. Embo J.

[B24] Taddei L, Chiarugi P, Brogelli L, Cirri P, Magnelli L, Raugei G, Ziche M, Granger HJ, Chiarugi V, Ramponi G (1999). Inhibitory effect of full-length human endostatin on in vitro angiogenesis. Biochem Biophys Res Commun.

[B25] Dhanabal M, Ramchandran R, Waterman MJ, Lu H, Knebelmann B, Segal M, Sukhatme VP (1999). Endostatin induces endothelial cell apoptosis. J Biol Chem.

[B26] Sauter BV, Martinet O, Zhang WJ, Mandeli J, Woo SL (2000). Adenovirus-mediated gene transfer of endostatin in vivo results in high level of transgene expression and inhibition of tumor growth and metastases. Proc Natl Acad Sci U S A.

[B27] Perletti G, Concari P, Giardini R, Marras E, Piccinini F, Folkman J, Chen L (2000). Antitumor activity of endostatin against carcinogen-induced rat primary mammary tumors. Cancer Res.

[B28] Blezinger P, Wang J, Gondo M, Quezada A, Mehrens D, French M, Singhal A, Sullivan S, Rolland A, Ralston R, Min W (1999). Systemic inhibition of tumor growth and tumor metastases by intramuscular administration of the endostatin gene. Nat Biotechnol.

[B29] Lochter A, Bissell MJ (1995). Involvement of extracellular matrix constituents in breast cancer. Semin Cancer Biol.

[B30] Noel A, Kebers F, Maquoi E, Foidart JM (1999). Cell-cell and cell-matrix interactions during breast cancer progression. Curr Top Pathol.

[B31] Nor JE, Christensen J, Mooney DJ, Polverini PJ (1999). Vascular endothelial growth factor (VEGF)-mediated angiogenesis is associated with enhanced endothelial cell survival and induction of Bcl-2 expression. Am J Pathol.

[B32] Nor JE, Christensen J, Liu J, Peters M, Mooney DJ, Strieter RM, Polverini PJ (2001). Up-Regulation of Bcl-2 in microvascular endothelial cells enhances intratumoral angiogenesis and accelerates tumor growth. Cancer Res.

[B33] Feng X, Clark RA, Galanakis D, Tonnesen MG (1999). Fibrin and collagen differentially regulate human dermal microvascular endothelial cell integrins: stabilization of alphav/beta3 mRNA by fibrin1. J Invest Dermatol.

[B34] Kanda S, Tomasini-Johansson B, Klint P, Dixelius J, Rubin K, Claesson-Welsh L (1999). Signaling via fibroblast growth factor receptor-1 is dependent on extracellular matrix in capillary endothelial cell differentiation. Exp Cell Res.

[B35] Isik FF, Gibran NS, Jang YC, Sandell L, Schwartz SM (1998). Vitronectin decreases microvascular endothelial cell apoptosis. J Cell Physiol.

[B36] Luscinskas FW, Lawler J (1994). Integrins as dynamic regulators of vascular function. Faseb J.

[B37] Ingber DE (1992). Extracellular matrix as a solid-state regulator in angiogenesis: identification of new targets for anti-cancer therapy. Semin Cancer Biol.

[B38] Thorburn A (2004). Death receptor-induced cell killing. Cell Signal.

[B39] Bantel H, Engels IH, Voelter W, Schulze-Osthoff K, Wesselborg S (1999). Mistletoe lectin activates caspase-8/FLICE independently of death receptor signaling and enhances anticancer drug-induced apoptosis. Cancer Res.

[B40] Inman GJ, Allday MJ (2000). Apoptosis induced by TGF-beta 1 in Burkitt's lymphoma cells is caspase 8 dependent but is death receptor independent. J Immunol.

[B41] Vogel T, Guo NH, Krutzsch HC, Blake DA, Hartman J, Mendelovitz S, Panet A, Roberts DD (1993). Modulation of endothelial cell proliferation, adhesion, and motility by recombinant heparin-binding domain and synthetic peptides from the type I repeats of thrombospondin. J Cell Biochem.

[B42] Galvin NJ, Vance PM, Dixit VM, Fink B, Frazier WA (1987). Interaction of human thrombospondin with types I-V collagen: direct binding and electron microscopy. J Cell Biol.

[B43] Volpert OV, Zaichuk T, Zhou W, Reiher F, Ferguson TA, Stuart PM, Amin M, Bouck NP (2002). Inducer-stimulated Fas targets activated endothelium for destruction by anti-angiogenic thrombospondin-1 and pigment epithelium-derived factor. Nat Med.

[B44] Jackson CJ, Nguyen M (1997). Human microvascular endothelial cells differ from macrovascular endothelial cells in their expression of matrix metalloproteinases. Int J Biochem Cell Biol.

[B45] Geiger M, Stone A, Mason SN, Oldham KT, Guice KS (1997). Differential nitric oxide production by microvascular and macrovascular endothelial cells. Am J Physiol.

[B46] Mason JC, Yarwood H, Sugars K, Haskard DO (1997). Human umbilical vein and dermal microvascular endothelial cells show heterogeneity in response to PKC activation. Am J Physiol.

[B47] Luster AD, Greenberg SM, Leder P (1995). The IP-10 chemokine binds to a specific cell surface heparan sulfate site shared with platelet factor 4 and inhibits endothelial cell proliferation. J Exp Med.

[B48] Yang J, Richmond A (2004). The angiostatic activity of interferon-inducible protein-10/CXCL10 in human melanoma depends on binding to CXCR3 but not to glycosaminoglycan. Mol Ther.

[B49] Sbaa-Ketata E, Vasse M, Lenormand B, Schneider P, Soria C, Vannier JP (2001). Fibronectin increases the migration induced by stromal cell-derived factor-1 alpha (SDF-1 alpha) in pre-B acute lymphoblastic leukemia cells. Eur Cytokine Netw.

[B50] Dixelius J, Larsson H, Sasaki T, Holmqvist K, Lu L, Engstrom A, Timpl R, Welsh M, Claesson-Welsh L (2000). Endostatin-induced tyrosine kinase signaling through the Shb adaptor protein regulates endothelial cell apoptosis. Blood.

[B51] Kim YM, Jang JW, Lee OH, Yeon J, Choi EY, Kim KW, Lee ST, Kwon YG (2000). Endostatin inhibits endothelial and tumor cellular invasion by blocking the activation and catalytic activity of matrix metalloproteinase. Cancer Res.

[B52] Wickstrom SA, Veikkola T, Rehn M, Pihlajaniemi T, Alitalo K, Keski-Oja J (2001). Endostatin-induced modulation of plasminogen activation with concomitant loss of focal adhesions and actin stress fibers in cultured human endothelial cells. Cancer Res.

[B53] Ergun S, Kilic N, Wurmbach JH, Ebrahimnejad A, Fernando M, Sevinc S, Kilic E, Chalajour F, Fiedler W, Lauke H, Lamszus K, Hammerer P, Weil J, Herbst H, Folkman J (2001). Endostatin inhibits angiogenesis by stabilization of newly formed endothelial tubes. Angiogenesis.

[B54] Dhanabal M, Volk R, Ramchandran R, Simons M, Sukhatme VP (1999). Cloning, expression, and in vitro activity of human endostatin. Biochem Biophys Res Commun.

[B55] Karumanchi SA, Jha V, Ramchandran R, Karihaloo A, Tsiokas L, Chan B, Dhanabal M, Hanai JI, Venkataraman G, Shriver Z, Keiser N, Kalluri R, Zeng H, Mukhopadhyay D, Chen RL, Lander AD, Hagihara K, Yamaguchi Y, Sasisekharan R, Cantley L, Sukhatme VP (2001). Cell surface glypicans are low-affinity endostatin receptors. Mol Cell.

[B56] Sasaki T, Larsson H, Kreuger J, Salmivirta M, Claesson-Welsh L, Lindahl U, Hohenester E, Timpl R (1999). Structural basis and potential role of heparin/heparan sulfate binding to the angiogenesis inhibitor endostatin. Embo J.

[B57] MacDonald NJ, Shivers WY, Narum DL, Plum SM, Wingard JN, Fuhrmann SR, Liang H, Holland-Linn J, Chen DH, Sim BK (2001). Endostatin binds tropomyosin. A potential modulator of the antitumor activity of endostatin. J Biol Chem.

[B58] Kim YM, Hwang S, Pyun BJ, Kim TY, Lee ST, Gho YS, Kwon YG (2002). Endostatin blocks VEGF-mediated signaling via direct interaction with KDR/Flk-1. J Biol Chem.

[B59] Rehn M, Veikkola T, Kukk-Valdre E, Nakamura H, Ilmonen M, Lombardo C, Pihlajaniemi T, Alitalo K, Vuori K (2001). Interaction of endostatin with integrins implicated in angiogenesis. Proc Natl Acad Sci U S A.

[B60] Wickstrom SA, Alitalo K, Keski-Oja J (2002). Endostatin associates with integrin alpha5beta1 and caveolin-1, and activates Src via a tyrosyl phosphatase-dependent pathway in human endothelial cells. Cancer Res.

[B61] Calvete JJ (2004). Structures of integrin domains and concerted conformational changes in the bidirectional signaling mechanism of alphaIIbbeta3. Exp Biol Med (Maywood).

[B62] Wickstrom SA, Alitalo K, Keski-Oja J (2004). An endostatin-derived peptide interacts with integrins and regulates actin cytoskeleton and migration of endothelial cells. J Biol Chem.

[B63] Stupack DG, Puente XS, Boutsaboualoy S, Storgard CM, Cheresh DA (2001). Apoptosis of adherent cells by recruitment of caspase-8 to unligated integrins. J Cell Biol.

[B64] Furumatsu T, Yamaguchi N, Nishida K, Kawai A, Kunisada T, Namba M, Inoue H, Ninomiya Y (2002). Endostatin Inhibits Adhesion of Endothelial Cells to Collagen I via alpha(2)beta(1) Integrin, a Possible Cause of Prevention of Chondrosarcoma Growth. J Biochem (Tokyo).

[B65] Wickstrom SA, Alitalo K, Keski-Oja J (2003). Endostatin associates with lipid rafts and induces reorganization of the actin cytoskeleton via down-regulation of RhoA activity. J Biol Chem.

[B66] Lohler J, Timpl R, Jaenisch R (1984). Embryonic lethal mutation in mouse collagen I gene causes rupture of blood vessels and is associated with erythropoietic and mesenchymal cell death. Cell.

